# The Long Life of Birds: The Rat-Pigeon Comparison Revisited

**DOI:** 10.1371/journal.pone.0024138

**Published:** 2011-08-31

**Authors:** Magdalene K. Montgomery, A. J. Hulbert, William A. Buttemer

**Affiliations:** 1 Diabetes and Obesity Program, Garvan Institute of Medical Research, Darlinghurst, New South Wales, Australia; 2 School of Biological Sciences, University of Wollongong, Wollongong, New South Wales, Australia; 3 Centre for Integrative Ecology, Deakin University, Waurn Ponds, Victoria, Australia; University of Queensland, Australia

## Abstract

The most studied comparison of aging and maximum lifespan potential (MLSP) among endotherms involves the 7-fold longevity difference between rats (MLSP 5y) and pigeons (MLSP 35y). A widely accepted theory explaining MLSP differences between species is the oxidative stress theory, which purports that reactive oxygen species (ROS) produced during mitochondrial respiration damage bio-molecules and eventually lead to the breakdown of regulatory systems and consequent death. Previous rat-pigeon studies compared only aspects of the oxidative stress theory and most concluded that the lower mitochondrial superoxide production of pigeons compared to rats was responsible for their much greater longevity. This conclusion is based mainly on data from one tissue (the heart) using one mitochondrial substrate (succinate). Studies on heart mitochondria using pyruvate as a mitochondrial substrate gave contradictory results. We believe the conclusion that birds produce less mitochondrial superoxide than mammals is unwarranted.

We have revisited the rat-pigeon comparison in the most comprehensive manner to date. We have measured superoxide production (by heart, skeletal muscle and liver mitochondria), five different antioxidants in plasma, three tissues and mitochondria, membrane fatty acid composition (in seven tissues and three mitochondria), and biomarkers of oxidative damage. The only substantial and consistent difference that we have observed between rats and pigeons is their membrane fatty acid composition, with rats having membranes that are more susceptible to damage. This suggests that, although there was no difference in superoxide production, there is likely a much greater production of lipid-based ROS in the rat.

We conclude that the differences in superoxide production reported previously were due to the arbitrary selection of heart muscle to source mitochondria and the provision of succinate. Had mitochondria been harvested from other tissues or other relevant mitochondrial metabolic substrates been used, then very different conclusions regarding differences in oxidative stress would have been reached.

## Introduction

As a group, birds are long-living with their maximum lifespan potential (MLSP) being on average twice that of similar-sized mammals [1], [2] and it can be much greater for some individual comparisons. The most common mammal-bird comparison in the scientific literature is the rat-pigeon comparison. The rat has a MLSP of 5 y, compared to 35 y for the similar-sized pigeon (both from the AnAge database: genomics.senescence.info). This seven-fold MLSP difference has the potential to give considerable insight into the processes that determine longevity. Importantly, this is many times the longevity difference generally achieved either by genetic manipulation or environmental manipulation (such as dietary restriction).

Early attempts to understand the mechanisms determining maximum longevity of different mammals implicated differences in metabolic rate (MR) [3], [4]. As well, studies with ectotherms showed that high temperatures reduced longevity [3]. Thus, the bird-mammal longevity difference was at first surprising as (i) the metabolic rate of birds is generally higher and, (ii) birds usually have higher body temperatures than mammals. Indeed, pigeons have both a higher body temperature than rats (∼41°C vs. ∼37°C) and a basal metabolic rate slightly greater than rats [5], [6].

While differences in MR *per se* cannot fully explain MLSP differences, there does appear to be some link between the ‘rate of living’ and the ‘length of life’ (see [1]). In the 1950's, the linkage of oxygen toxicity to free radical production [7] prompted the “free radical” theory of aging [8] which proposed that reactive oxygen species (ROS), produced as a normal by-product of metabolism, cause oxidative damage to biological molecules and the accumulated damage, in turn, results in the breakdown of homeostasis, eventually causing death and consequently determining the characteristic maximum longevity of each particular species. Over the years the free radical theory has morphed into the “oxidative stress” theory of aging (sometimes also called the oxidative damage theory) [1]. This theory is currently the most widely accepted explanation of aging and thus MLSP determination. It can be divided into its functional components, (i) the mitochondrial production of ROS, (ii) the countervailing influence of antioxidant systems (both enzymatic and non-enzymatic), and (iii) oxidative damage to bio-molecules. Although there is much evidence to support the oxidative stress theory, a number of recent contributions have questioned it's general applicability [9], [10], [11]. A recent modification of the theory, the “membrane pacemaker” theory [12], emphasises that species vary in their membrane fatty acid composition and proposes that this difference in membrane composition (especially membrane polyunsaturated fatty acids) has significant flow-on effects and may be an important determinant of maximum longevity. It emphasises that a large number of powerful lipid-based ROS are secondarily produced by the action of primary ROS (superoxide, hydrogen peroxide, hydroxyl radicals) on membrane lipids. This differentiation between production of primary ROS and secondary ROS will be more fully described in the discussion.

We have found 15 reports in the literature where aspects of the oxidative stress theory have been reported for both rats and pigeons in the same study with 53% comparing antioxidants, 40% comparing mitochondrial ROS production, 40% comparing oxidative damage, and 20% comparing membrane fatty acid composition. To our knowledge the earliest comparison is that of Matkovics et al. [13] which determined the activity of antioxidant enzymes. It showed that the activity (per g tissue) of superoxide dismutase and peroxidase was much greater (2-fold on average), while catalase activity was much less in pigeons compared to rats. Two groups (the Barja group in Spain and the Sohal group in the U.S.A.) were responsible for a number of seminal rat-pigeon studies in the early 1990s, and their results agreed with the Matkovics findings in some tissues, but not in others [14], [15]. Similarly, more recent rat-pigeon antioxidant studies report a variety of contradictory results [16], [17]. Early perspectives on antioxidant comparisons hypothesised that longer-living species should have higher levels of antioxidant defences than shorter-living species. The common finding of either the opposite result, or no correlation has resulted in the more recent hypothesis that high antioxidant defences are associated with high oxidative stress [18], [19]. Indeed, high antioxidant enzyme activities are now often interpreted to reflect a high level of oxidative stress.

Later, several studies compared the *‘in vitro’* production of ROS by isolated cardiac mitochondria and demonstrated that those from the shorter-living rat have higher rates of superoxide and H_2_O_2_ formation than pigeon heart mitochondria with succinate, but not with pyruvate as the substrate [16], [20], [21]. Only one study reports higher ROS production in rat heart mitochondria with pyruvate as substrate [22]. ROS production has also been compared in liver, brain, lung and kidney mitochondria and these studies similarly report a higher ROS production in the rat only when succinate was used as mitochondrial substrate [15], [20]. The extrapolation of these rat-pigeon findings to the common generalization that bird mitochondria produce less ROS than mammals should be treated with caution. In our opinion, one major concern is whether inter-specific comparisons of mitochondrial rates “per mg mitochondrial protein” are physiologically appropriate.

Comparison of mitochondrial function on a “per mg mitochondrial protein” basis is the most common denominator used in scientific studies and, while it is quite adequate to examine the effect of a treatment on mitochondria from a specific source, it might be an inappropriate denominator when comparing mitochondria either from different tissues or from different species [23]. The same qualification also holds when enzyme activities are compared on a “per mg tissue protein” basis because of possible variation in the relative protein content of different tissues from different species. A more appropriate denominator for our current comparison is to express mitochondrial ROS production on a “per g tissue” (or where possible on a “per whole tissue” basis). In this contribution, we express ‘*in vitro’* ROS production on both a “per mg mitochondrial protein” basis and on a “per g tissue” basis (as well as “per whole tissue” for heart and liver).

There are fewer comparisons of oxidative damage in rats and pigeons than antioxidant and mitochondrial ROS comparisons. Some report higher oxidative damage in rats compared to pigeons [14], [24], [25], [26], some report no difference [26], [27], while others report less oxidatively damaged products [28]. Similarly, there are rat-pigeon comparisons of membrane fatty acid composition. As only polyunsaturated fatty acids are peroxidised by mitochondrial ROS, it is possible, knowing their fatty acid composition, to calculate a peroxidation index (PI) which represents the susceptibility of membrane lipids to oxidative damage [29]. It has been previously reported, for both heart and liver, that pigeons have a membrane composition less susceptible to peroxidative damage than rats [25], [26].

In view of the contradictory findings of previous rat-pigeon comparisons and as part of a larger comparison among bird species that differ significantly in maximum longevity, we decided to revisit the rat-pigeon comparison. Here we report results from the most extensive individual examination of the oxidative stress theory as an explanation for the seven-fold MLSP difference between pigeons and rats. In tissues obtained from healthy young adults, we have measured: (i) the levels of seven antioxidant systems (both enzymatic and non-enzymatic) in up to four tissues at both the total tissue and mitochondrial levels, (ii) the ‘*in vitro*’ production of primary ROS (superoxide and hydrogen peroxide) by mitochondria isolated from three tissues, when provided with two different substrates, (iii) the fatty acid composition and calculated susceptibility to oxidative damage of membrane lipids from seven different tissues, as well as mitochondrial membrane lipids from three tissues, and (iv) the concentrations of four products of oxidative damage (including protein, mitochondrial DNA and lipid damage) in up to four tissues. We conclude from this comparison that some, but not all components of the “oxidative stress” theory support the notion that the relatively long life of pigeons compared to rats is associated with a lower level of oxidative stress in pigeon tissues. Our results suggest that differences in the production of primary ROS (superoxide and hydrogen peroxide) may not be as important as production of secondary ROS (especially lipid-based ROS).

## Materials and Methods

### Animals and Tissue Sampling

All experiments were approved by the University of Wollongong Animal Ethics Committee and were conducted in conformity with the NHMRC Australian Code of Practice for the Care and Use of Animals for Scientific Purposes. Eight pigeons were from a local breeder (Andrew's Pet Shop, Smithfield, Australia) and were of mixed sex. Six male Wistar rats (*Rattus norvegicus)* were purchased from the Australian Resource Centre, Canning Vale, Australia. All animals had free access to commercial diets and water. Rats were kept in a facility maintained at 25°C and a 12h:12h light:dark cycle whereas the pigeons were housed in outdoor aviaries (ca. 13h:11h light:dark; 18–26°C). We chose animals of the same chronological age, approximately six months old, rather than biological age, with the expectation that more rapidly aging species will have higher overall oxidative stress and greater accumulation of oxidative-damage biomarkers than species that age more slowly.

Following euthanasia using carbon dioxide, aliquots of liver, pectoral muscle and heart were transferred to cryovials and stored in liquid nitrogen for subsequent analyses. Blood was collected into heparinised vials following cardiac puncture and centrifuged to extract plasma, which was then stored in liquid nitrogen prior to later analyses. Separate aliquots of selected tissues were placed in buffer solutions for mitochondrial extraction (see [30]) prior to determining mitochondrial ROS production (see below).

### Chemicals

Amplex Ultra Red was purchased from Invitrogen, Mount Waverley, Australia. Black 96-well-plates were purchased from Interpath Services, Caringbah, Australia. Quantichrom Glutathione and Uric Acid Assay Kits were purchased from BioCore, Alexandria, Australia. Lipid Hydroperoxide, 8-hydroxy-2-deoxy Guanosine EIA and Catalase Assay Kits were purchased from Sapphire Bioscience, Redfern, Australia. The Wizard SV Genomic DNA Purification Kit was purchased from Promega, Alexandria, Australia. All solvents used for phospholipid extraction were from Crown Scientific, Moorebank, Australia. All other chemicals were from Sigma-Aldrich, Castle Hill, Australia.

### Isolation of mitochondria

Heart, pectoral muscle and liver mitochondria were isolated by differential centrifugation as described previously [30]. The protein concentration of mitochondrial suspensions was determined by the Biuret method using bovine BSA as a standard [31].

Briefly, for pectoral muscle and heart mitochondria, the tissue was finely diced in CP-1 medium (100 mM KCl, 50 mM Tris/HCl, pH 7.4, and 2 mM EGTA), digested on ice for 3 min in CP-2 medium [CP-1, to which was added 0.5% (w/v) BSA, 5 mM MgCl_2_, 1 mM ATP and 2.45 units ml–1 Protease Type VIII (Sigma P 5380)] and homogenized using a dounce homogenizer. The homogenate was spun for 10 min at 500 *g* and 4°C. The resulting supernatant was subjected to a high-speed spin cycle (10 600 *g*, 10 min, 4°C) and the pellet was re-suspended in CP-1. The high-speed spin cycle was repeated and the re-suspension finally centrifuged for 10 min at 3800 *g* and 4°C. The final pellet was re-suspended in a minimum volume of CP-1 buffer.

For the isolation of liver mitochondria, the liver was immediately placed in ice-cold STE buffer (250 mM sucrose, 5 mM Tris/HCl, pH 7.4, and 2 mM EGTA), minced with scissors and disrupted with a dounce homogenizer. The homogenate was spun for 3 min at 1000 *g* and 4°C, and the supernatant centrifuged for 10 min at 10 600 *g* and 4°C. The high-speed spin cycle was repeated twice and the final pellet re-suspended in a minimal volume of isolation medium.

### Mitochondrial Respiration (Respiratory control ratio)

Mitochondrial oxygen consumption was determined as described previously [30]. Oxygen consumption was measured using a Clarke-type electrode (Rank Brothers Ltd, Cambridge, UK) maintained at 37°C for the rats and at 41°C for the pigeons and calibrated with air-saturated medium [120 mM KCl, 5 mM K_2_HPO_4_, 3 mM Hepes, 1 mM EGTA, 0.3% (w/v) defatted BSA, 7 µM rotenone (to inhibit complex I of the respiratory chain), adjusted to pH 7.2], which was assumed to contain 406/381 nmol oxygen/ml, respectively [32]. Mitochondrial respiration was started by adding 5 mM succinate (state 2 respiration). The respiratory control ratio (RCR), determined by dividing state 3 respiration (addition of 800 µM ADP to achieve maximum oxygen consumption) by state 4 respiration (5 µg/ml oligomycin, which inhibits the F1Fo-ATP synthase and prevents ATP synthesis), was measured to ascertain the functional integrity of the mitochondria.

### Mitochondrial H_2_O_2_ production

Superoxide production in isolated mitochondria was determined indirectly by monitoring the oxidation of homovanillic acid or Amplex Ultra Red as described previously [21], [33]. H_2_O_2_ was detected using a BMG Labtech FLUOstar OPTIMA fluorescence plate reader. Rat and pigeon mitochondria were incubated at 0.5 mg/ml at 37°C or 41°C, respectively, in assay medium (120 mM KCl, 3 mM Hepes, 1 mM EGTA, 0.3% BSA, pH 7.2 at 37°C or 41°C, respectively) containing 6 u/ml horseradish peroxidase (HRP, One pyrogallol unit will form 1.0 mg purpurogallin from pyrogallol in 20 sec at pH 6.0 and 20°C), 30 u/ml superoxide dismutase (SOD, One unit will inhibit reduction of cytochrome c by 50% in a coupled system with xanthine oxidase at pH 7.8 and 25°C in a 3.0 ml reaction volume), and Amplex Ultra Red (0.1 mM) or homovanillic acid (HVA, 0.2 mM). Appropriate fluorophor concentrations were determined through a stepwise calibration of the plate reader using known quantities of H_2_O_2_.

Mitochondrial ROS production was determined under 3 different conditions: (1) at state 2 (no inhibitors), (2) at state 4 (1 µg/ml oligomycin), (3) and at state 4 adding rotenone (2 µM). Rotenone inhibits complex I of the respiration chain and prevents a backflow of electrons in succinate-supplemented mitochondria. After a 5-min incubation period at 37°C or 41°C inside the plate reader, ROS production was started by injecting (using on-board injectors) a substrate for the mitochondrial respiration chain: either pyruvate and malate (both 2.5 mM) or succinate (5 mM). ROS production, observable as a linear increase in fluorescence, was monitored for 10 minutes.

### Calibrations and Corrections

Calibrations and corrections were carried out as described elsewhere [33]. The presence of mitochondria had no effect on the calibration (addition of known amounts of H_2_O_2_) using Amplex Ultra Red, but quenched the fluorescence using HVA; the slopes with liver, heart and pectoral muscle mitochondria were 72, 86 and 86% in rats and 73, 95 and 91% in pigeons, respectively, of the corresponding slopes of control curves without mitochondria. Horseradish peroxidase and superoxide dismutase were used in excess as a further addition of either enzyme did not affect the results (data not shown). Control measurements were carried out in the absence of mitochondria, HVA or Amplex Ultra Red. With Amplex Ultra Red, fluorescence signals remained constant. With HVA, dependent on the absence or presence of inhibitors, control measurements led to a slight linear increase or decrease in fluorescence, respectively. The final rates of H_2_O_2_ production, as shown in the results section, have been fully corrected by subtracting background rates associated with control measurements.

### Free Radical Leak

Production of primary ROS (in the absence and presence of rotenone) and state 4 respiration rates of succinate-supplemented mitochondria were used to calculate the percentage of electrons which leak out of sequence producing superoxide and subsequently hydrogen peroxide in succinate-supplemented mitochondria [9]. Whereas two electrons are needed for the reduction of 1 mol of O_2_ to H_2_O_2_, four electrons are transferred in the reduction of 1 mol of O_2_ to water. Therefore, the percent free radical leak was calculated as the rate of H_2_O_2_ production divided by twice the rate of oxygen consumption, and the result was multiplied by 100 [34]. Values for primary ROS production and oxygen consumption expressed “per mg of mitochondrial protein” were used to calculate the FRL.

### Cytochrome c oxidase

Cytochrome c (COX, 0.3%) was reduced in phosphate buffer (10 mM KH_2_PO_4_, pH 7.2 at 25°C, 0.1 mM EDTA) containing Na_2_S_2_O_4_, and aerated for 20 min. 860 µl of phosphate buffer was mixed with 120 µl of the reduced cytochrome c and 20 µl of sample and the decrease in absorbance was determined at 550 nm and 37°C. Cytochrome c activity was determined in whole tissue homogenates and in mitochondrial preparations of the same tissues. Knowing, 1) the amount of mitochondrial protein per unit COX in the pellet, and 2) the COX units per g tissue, we calculated the amount of mitochondrial protein per g tissue.

### Total antioxidant status

Total antioxidants in plasma were determined using two different assays: the total antioxidant status (TAS) assay and the ferric reducing ability of plasma (FRA) assay. The TAS assay was carried out as described previously [35] using a temperature-controlled spectrophotometer (Varian Cary 300) at 25°C. Trolox (0.2 mM–1 mM), a vitamin E derivative, was used as an antioxidant standard and results are shown in mM Trolox equivalents.

Total antioxidant status in heart, pectoral muscle and liver were determined with the FRA assay. Tissues were homogenised in buffer (100 mM KH_2_PO_4_, 1 mM EDTA) and a FRA reagent was prepared as described elsewhere [36]. Blank, standards or samples were mixed with the reagent and, after a 4-min incubation, the absorbance was determined at 593 nm and 37°C using a temperature-controlled spectrophotometer (Varian Cary 300). Ferrous sulphate was used as a standard and the results are expressed in mM Fe^2+^.

### Enzymatic antioxidants

Superoxide dismutase (SOD), glutathione peroxidase (GPx) and catalase (CAT) content were determined in plasma, tissue homogenates and mitochondrial extractions. Tissues were homogenised with an Ultra Turrax (11,000/min, 15 sec) in buffer (SOD: 210 mM mannitol, 70 mM sucrose, 20 mM Hepes, 1 mM EGTA, pH = 7.5 at 25°C) (GPx: 50 mM Tris-HCl, 5 mM EDTA, 1 mM Dithiothreitol, pH = 7.5 at 25°C) (CAT: 50 mM KH_2_PO_4_, 1 mM EDTA, pH = 6.7 at 25°C). Homogenates were centrifuged at 4°C and 1,500 g for 5 min (SOD) or at 10,000 g for 15 min (GPx, CAT) and the supernatant used for the assay.

SOD activity was measured as the inhibition of the rate of cytochrome c reduction by superoxide which was followed at 550 nm and 25°C using a temperature-controlled spectrophotometer (Varian Cary 300), adapted from a method described elsewhere [37]. Briefly, a reaction cocktail was prepared by mixing 11.5 ml of distilled water, 12.5 ml of buffer (216 mM KH_2_PO_4_, pH = 7.8 at 25°C), 0.5 ml EDTA (10.7 mM), 0.5 ml cytochrome c (1.1 mM) and 25 ml xanthine (0.108 mM). 265 ul of the reaction cocktail was mixed with 10 ul blank (distilled water), standard or sample. 25 µl of xanthine oxidase (0.05 u/ml) was added and the increase in absorbance was recorded for 5 min at 550 nm and 25°C.

GPx activity was determined indirectly as the decrease in NADPH absorption at 340 nm and 25°C, as described elsewhere [38]. A reaction cocktail was prepared by mixing azide buffer (9.2 ml, 50 mM NaH_2_PO_4_, 0.4 mM EDTA, pH = 7.0 at 25°C, addition of 1 mM Na-azide), glutathione reductase (100 µl, 100 u/ml) and GSH (50 µl, 200 mM) into a 1 mg beta-NADPH vial. The reaction cocktail was mixed with blank, standard or sample and H_2_O_2_ (5 µl, 0.042% (v/v)) was added to start the reaction. The decrease in absorbance was monitored for 5 min. GPx standards were diluted in 10 mM NaH_2_PO_4_, 1 mM DTT, pH = 7.0 at 25°C.

Catalase was measured in plasma, liver and heart of all animals using the catalase assay kit from Cayman Chemicals (Sapphire Bioscience, Redfern, Australia). The catalase assay was carried out at 25°C as per manufacturer's instructions.

### Non-enzymatic antioxidants

Plasma uric acid levels were determined using a commercially available kit (QuantiChrom™ Uric Acid Assay Kit, Bioassay Systems). GSH levels were determined in blood, heart and liver using a commercially available kit (QuantiChrom™ Glutathione Assay Kit, Bioassay Systems). Tissues were homogenised with an Ultra Turrax (11,000/min, 15 sec) in 50 mM KH_2_PO_4_, 1 mM EDTA, pH = 6.7. Tissue homogenates were centrifuged for 15 min at 10,000 g and 4°C, and the supernatant used for the assay.

### Fatty acid extraction

Lipids were extracted, phospholipids were isolated and the fatty acid composition of those phospholipids was determined by gas chromatography. Briefly, all solvents used contained 0.01% (w/v) butylated hydroxytoluene. Tissues (0.1–0.2 g) and mitochondria were homogenised in 4–5 ml of a 2∶1 (v/v) chloroform-methanol mixture, and rotated at 4°C overnight. Sulphuric acid (2 ml of 1 M solution) was added, the samples spun for 10 min at 1000 rpm, and the chloroform layer transferred into new tubes. This step was repeated a second time, followed by the addition of sodium hydrosulphite and a filtration step using a Pasteur pipette containing silane treated glass wool. The extracted lipids were dried down under nitrogen following an addition of 5 ml Hexane. Phospholipids were separated from total lipids using Sep-Pak Classic Silica Cartridges (Waters, Rydalmere, Australia). This separation step included trapping of the triglycerides and phospholipids on the column, elution of the triglycerides using ethyl acetate, and the final elution of phospholipids using methanol (3×5 ml). Methanol was removed by a drying down step under nitrogen, followed by the addition 2 ml of a 4∶1 (v/v) methanol-toluene mixture and a transmethylation step using 200 µl acetyl chloride. The samples were heated to 100°C for 1 h, cooled on ice and 5 ml of a 6% K_2_CO_3_ solution was added. The tubes were vortexed and centrifuged for 10 min at 3000 rpm. The upper toluene phase containing the phospholipids was transferred into GC vials.

The extracted fatty acid methyl esters were measured by gas chromatography (Shimadzu GC-17A, Rydalmere, Australia) using a Varian WCOT fused silica column (50 m × 0.25 mm internal diameter, CP7419, Sydney, Australia) with the following temperature program: 150°C initial temperature; 17.5°C/min to 170°C; 0.5°C/min to 178°C; 15°C/min to 222°C; 2°C/min to 232°C. Fatty acid composition of tissues and mitochondria was identified by comparison with an external standard (FAME Mix C4-C24; Sigma Aldrich, Sydney, Australia) and expressed as mole percentage of total fatty acids. The peroxidation index is a measure of the calculated susceptibility of the phospholipid fatty acids to peroxidative damage and was calculated as PI  =  (0.025 × % monoenoics) + (1 × % dienoics) + (2 × % trienoics) + (4 × % tetraenoics) + (6 × % pentaenoics) + (8 × % hexaenoics) [29].

### Measures of lipid peroxidation

Lipid hydroperoxides were determined in plasma and liver using a Lipid Hydroperoxide Assay Kit from Cayman Chemicals (Sapphire Bioscience, Redfern, Australia). The TBARS (thiobarbituric acid reactive substances) assay was used as a measure of lipid peroxidation. Tissues were homogenised in buffer (100 mM KH_2_PO_4_, 1 mM EDTA). Blank, standard (tetramethoxypropane) or samples were mixed with butylated hydroxytoluene (BHT, 3 mM) and thiobarbituric acid (TBA, 0.4% (w/v) in 10% acetic acid solution (v/v), pH 5.0), and incubated for one hour at 90°C. Butanol was added and the samples centrifuged for 10 min at 7500 rpm. The absorbance of the butanol phase was determined at 532 nm.

### Measure of protein damage – Protein carbonyls

Tissues were homogenised in buffer (50 mM KH_2_PO_4_, 1 mM EDTA, pH = 6.7) and the homogenate centrifuged for 15 min at 10,000 g and 4°C. Nucleic acids were removed using 1% streptomycin (incubation at room temperature for 15 min, followed by centrifugation at 6,000 g, 10 min, 4°C), and the supernatant used for the assay. Protein carbonyls were derivatized to 2,4-dinitrophenylhydrazone by reaction with 2,4-dinitrophenylhydrazine (DNPH) as described previously [39]. Briefly, 25 µl of sample were mixed with either 200 µl of DNPH (sample tube) (10 mM DNPH in 2.5 M HCl) or 200 µl 2.5 M HCl (control tube), followed by a 1-h incubation at room temperature in the dark. Trichloracetic acid (TCA, 250 µl, 20%) was added to each tube, the tubes vortexed, placed on ice for 5 min, and centrifuged for 10 min at 10000 g and 4°C. The pellet was re-suspended in TCA (250 µl, 10%), vortexed, placed on ice for 5 min and spun again. The previous step was repeated 3x, 2x using ethanol-ethyl acetate (250 µl, 1∶1), and the last time using guanidine hydrochloride (350 µl, 6 M). The absorbance of the supernatant was read at 370 nm. The mean absorbance of the control tubes was subtracted from the mean absorbance of the sample tubes and the extinction coefficient for DNPH (0.022/µM/cm) was used to calculate the protein carbonyl concentration.

### Measure of DNA damage – 8-OHdG

DNA damage was determined in heart and liver mitochondria using the 8-hydroxy-2-deoxy-guanosine (8-OHdG) EIA Kit from Cayman Chemicals (Sapphire Bioscience, Redfern, Australia). Briefly, mitochondria were isolated and mitochondrial DNA was extracted using a commercially available kit (Wizard SV Genomic DNA Purification System, Promega). The DNA was heat denatured at 95°C for 10 min and digested for 1 h at 50°C using nuclease P1 (43 ng/µg DNA, Sigma-Aldrich N8630). Nuclease P1 was diluted in acetate buffer (20 mM C_2_H_3_O_2_Na•3H_2_O, 5 mM ZnCl_2_, 50 mM NaCl, pH = 5.3 at 50°C). The pH of each sample was adjusted to 8.0 with 1 M Tris and alkaline phosphatase was added (1 u/100 µg DNA). The mixture was incubated at 37°C for 30 min, boiled for 10 min and then placed on ice. Samples were used directly in the 8-OH-dG assay.

### Statistical analysis

All results are given as mean ± standard error with n being the number of animals used in each assay. The significance of differences between means was assessed using Student's t-test. P-values of < 0.05 were taken to be significant. There was no effect of gender on any of the measurements made on pigeons, so we assume that the effect of gender is negligible for the parameters we measured, as similarly reported by others [40], [41], [42], [43].

## Results

### Tissue size and protein content in rats and pigeons

Pigeons have a 7-fold higher maximum lifespan than rats, but a similar BMR. Pigeons also have a much larger heart (∼5-fold larger), but a smaller liver (∼2-fold smaller) than the rat (Table 1). When we examined heart, skeletal muscle and liver tissues, we found that pigeon tissues contain more protein than the same amount of rat tissue (Table 1). Pigeons had less mitochondrial protein per g heart and liver (significant only for liver) than the rat, but significantly more mitochondrial protein per g skeletal muscle than the rat. Consequently, the percentage of total tissue protein that is mitochondrial protein is significantly lower in the heart and liver of the pigeon compared to the rat (Table 1). These rat-pigeon differences in protein content emphasise the vagaries of comparing variables relative to protein content in inter-specific comparisons.

**Table 1:** Maximum lifespan potential (MLSP), basal metabolic rate (BMR), body mass, organ sizes, and total and mitochondrial protein content of rat and pigeon.

Parameter	Tissue	Rat	Pigeon	Significance
MLSP (years)		5	35	
BMR (ml O_2_/g/h)		0.67	0.78	
Body mass (g)		343±7	417±21	*
Heart size (g)		1.0±0.0	5.0±0.4	**
Liver size (g)		4.6±1.1	6.4±0.4	**
Protein(mg total protein/g tissue)	Heart	160±15	290±19	**
	Muscle	230±36	460±25	**
	Liver	200±17	250±23	
Mitochondrial protein(mg mitochondrial protein/g tissue)	Heart	28.9±6.8	17.9±5.5	
	Muscle	6.7±1.3	18.9±4.1	*
	Liver	14.3±3.7	5.6±0.8	**
Mitochondrial protein(% of total protein)	Heart	16.8±3.8	6.2±2.1	*
	Muscle	3.6±1.0	4.4±1.0	
	Liver	5.9±0.4	2.6±0.5	**

MLSP was obtained from http://genomics.senescence.info. BMR data were taken from [6] (pigeon) and [5] (rat). All other parameters were measured in this study. Significant differences are highlighted with one (p<0.05) or two asterisks (p<0.01).

### Mitochondrial oxygen consumption in rat and pigeon tissues

In order to ascertain that extracted mitochondria were functionally intact, we measured mitochondrial respiration polarographically in the presence and absence of ADP while using succinate as substrate for the respiratory chain. All our mitochondrial preparations were coupled and thus functionally intact (Table 2). There was no significant rat-pigeon difference in respiratory control ratio for heart or skeletal muscle mitochondria but rat liver mitochondria were significantly more coupled than pigeon liver mitochondria. Although pigeon heart and liver mitochondria were measured at a higher temperature than the rat tissues (41°C versus 37°C, respectively) they had a significantly lower state 3 respiration rate than the equivalent rat mitochondria (Table 2).

**Table 2:** Oxygen consumption and respiratory control ratios of rat and pigeon heart, skeletal muscle and liver mitochondria.

	Rat	Pigeon	Significance
**State 3 Respiration**(nmol O_2_/min/mg mitochondrial protein)			
Heart	106.8±23.2	42.0±9.3	*
Skeletal muscle	42.9±6.7	62.1±12.0	
Liver	89.3±9.6	45.5±8.9	*
**State 4 Respiration**(nmol O_2_/min/mg mitochondrial protein)			
Heart	20.5±3.5	15.6±3.3	
Skeletal muscle	12.8±1.2	28.2±4.3	**
Liver	12.5±1.7	11.2±2.4	
**Respiratory Control Ratio**			
Heart	5.6±1.2	4.9±1.9	
Skeletal muscle	3.3±0.4	2.3±0.4	
Liver	8.2±1.5	4.9±0.3	*

Mitochondrial oxygen consumption was determined in the presence of 5 mM Succinate and 7 uM Rotenone. State 3 respiration was initiated through the addition of 800 uM ADP and state 4 respiration rates were obtained through the addition of 5 ug/ml oligomycin. Shown are means ±SEM, n = 7 for rats and n = 8 for pigeons. A significant difference between rats and pigeons is illustrated by one (p<0.05) or two asterisks (p<0.01).

The skeletal muscle commonly examined in pigeons (and birds in general) is the pectoral muscle, while in rats it is more commonly the hind-limb muscles (both for reasons of size). In this study, because we chose to compare the same skeletal muscles, we examined pectoral muscles in both species. Our values obtained for rat pectoral muscle (respiration, ROS production, antioxidant and oxidative stress biomarker levels) are very similar to those reported for rat hind limb muscle [44], [45], [46], [47], [48], [49]. Thus, we assume that rat pectoral muscle is representative of rat skeletal muscle in general.

### Mitochondrial production of primary ROS

**Figure 1:**
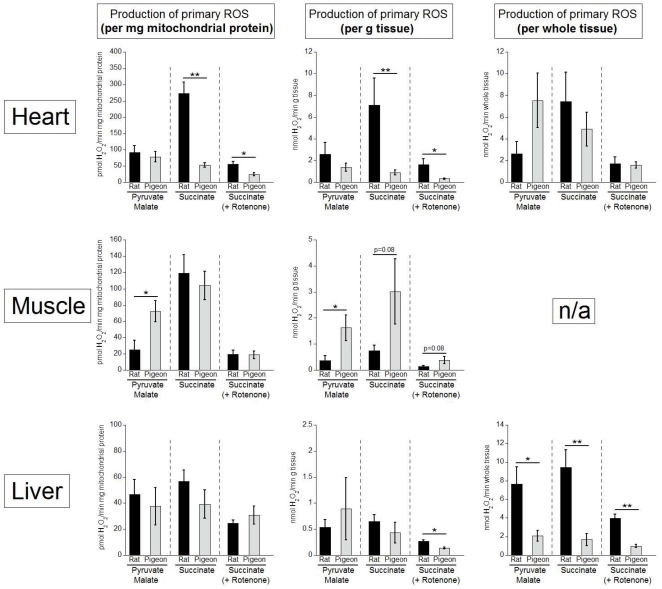
Mitochondrial production of primary reactive oxygen species (ROS). Primary ROS production was measured in rat (black bars) and pigeon (grey bars) heart, pectoral muscle and liver using pyruvate (+malate) and succinate as substrates for the respiration chain. Using succinate, ROS production was determined in the absence and presence of the complex I inhibitor rotenone. Primary ROS production is expressed in pmol H_2_O_2_/min/mg mitochondrial protein (left graphs), nmol H_2_O_2_/min/g tissue (middle graphs) and in nmol H_2_O_2_/min/whole tissue (right graphs). Shown are means ± SEM, n = 7 for rats and n = 8 for pigeons. Significant differences are highlighted with one (p<0.05) or two asterisks (p<0.01).

The ‘*in vitro’* production of superoxide and hydrogen peroxide by mitochondria isolated from heart, skeletal muscle and liver of rats and pigeons (using two different substrates) are presented in Figure 1. Because studies reporting ROS production commonly use different fluorescent reactants, we used both homovanillic acid and Amplex Ultra Red in separate assays of all mitochondria. After careful calibration and control for background fluorescence and fluorescent quenching (see [33]), both reactants gave identical results (data not shown). The values in Figure 1 are those obtained using Amplex Ultra Red.

The rates of primary ROS production by mitochondria are presented for both species in three forms: “per mg mitochondrial protein”, “per g tissue” and “per total tissue” (Fig. 1). The values shown were obtained from mitochondria under state 4 (non-phosphorylating) conditions, but identical results were obtained from mitochondria under state 2 conditions (data not shown). The first impression obtained from Figure 1 is that rat mitochondria show no consistent pattern of producing significantly more ROS than pigeon mitochondria. Indeed, on a “per mg mitochondrial protein” basis, it was only heart mitochondria that showed a significantly higher primary ROS production in rats compared to pigeons, and this was only when succinate was provided as a substrate for mitochondrial respiration. In skeletal muscle mitochondria, ROS production was significantly higher in pigeons compared to rats (the opposite of the hypothesised result), but once again only with one substrate (pyruvate) and not with the other (succinate). In all other cases, there was no significant difference between mitochondrial primary ROS production in pigeons and rats.

When calculated on a “per g tissue” basis, a similar pattern is evident, but with the addition that skeletal muscle mitochondria from pigeons had a higher primary ROS production with both substrates (Fig. 1). However, when expressed on a “per total tissue” basis a different picture emerges. There was no difference in the total ROS produced by the rat heart and pigeon heart while the total ROS produced by the rat liver was significantly greater than that produced by the pigeon liver.

Insight into tissue and species differences in sites of superoxide production along the electron transport chain (ETC) is revealed by comparing the effects of the two substrates on ROS production. Because succinate has the capacity to cause reverse electron flow from complex II towards complex I, a situation associated with high rates of ROS production (Fig. 1), the extent of ROS production associated with this reverse electron flow is revealed by comparing ROS production rates in the presence of rotenone, a potent inhibitor of complex I, to those with succinate alone. In all cases, complex I inhibition caused a substantial decrease in primary ROS production, particularly in muscle tissues (Fig. 1). Interestingly, the same pattern of tissue and species differences in ROS production rate with succinate occurred in the presence and absence of rotenone. Because rotenone restricts ROS production to mainly complex III [50], this means that complexes I and III produced ROS in a parallel fashion. We assume that the amount of rotenone (2 µM) used in the assay was high enough to fully inhibit complex I in both species as a ten-fold lower concentration used in similar conditions (0.2 µM) was enough to completely inhibit complex I [46].

### Primary ROS production relative to mitochondrial oxygen consumption

**Figure 2:**
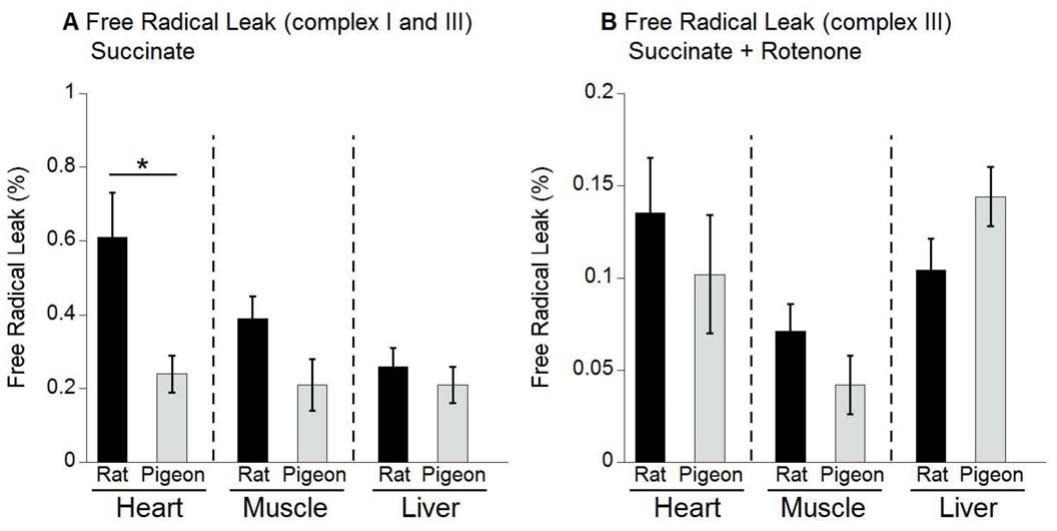
The mitochondrial free radical leak (FRL). The FRL was determined in the absence (A) and presence of rotenone (2 µM; B) in succinate-energized mitochondria of rat (black bars) and pigeon (grey bars) heart, pectoral muscle and liver mitochondria. The FRL is the percentage of electrons leaking out of sequence towards the production of superoxide. Rotenone inhibits complex I of the respiration chain; the FRL shown in (B) results mainly from electrons leaking at complex III. For FRL calculations see method section. Shown are means ± SEM, n = 7 for rats and n = 8 for pigeons. Significant differences are highlighted with an asterisk (p<0.05).

Another way of comparing mitochondrial production of primary ROS is to express it relative to the rate of mitochondrial oxygen consumption. Using reasonable assumptions, it is possible to convert both superoxide production and oxygen consumption to the same units (electrons) and thus calculate the percent of electrons that, rather than being transported to molecular oxygen as eventual electron acceptor, instead leak out of the respiratory chain and produce superoxide (for calculations see method section and [34]). This has been called the free radical leak (FRL). By combining the data in Figure 1 and Table 2, we are able to calculate FRL when mitochondria are respiring on succinate as substrate, both with and without rotenone. These results are presented in Figures 2A and 2B.

We assume that the FRL calculated from measurements using rotenone represent the FRL from complex III only, while those measured without rotenone include the combined FRL at complex III (from “forward electron transport”) and complex I (from “reverse electron transport”). Interestingly, the use of rotenone in this case may represent one of the few situations where use of an inhibitor provides a more physiologically relevant measure than not using the inhibitor. The presence of rotenone does not affect mitochondrial respiration when using succinate as a substrate [51], [52].

As can be seen from Figure 2B, there is no significant rat-pigeon difference in the calculated FRL from either heart, skeletal muscle or liver mitochondria in the presence of rotenone and using succinate as substrate, with all values in the range of 0.04% to 0.14%. Without rotenone (Fig 2A), there was no rat-pigeon difference in FRL calculated for skeletal muscle and liver mitochondria (values of 0.2% – 0.4%), but rat heart mitochondria had a significantly higher FRL compared to pigeon heart mitochondria (0.6% vs. 0.2%). Taken together, these results suggest that for heart mitochondria, it is “reverse electron transport” that is responsible for the significant rat-pigeon difference.

### Antioxidant defences in rats and pigeons

The plasma total antioxidant capacity did not differ between rats and pigeons, based on both the “total antioxidant status” (TAS) and the “ferric reducing ability” (FRA) assays (Table 3). The FRA assay was also used to determine total antioxidant capacity of tissue homogenates. Whereas total antioxidant capacity of heart and liver was the same in rats and pigeons, it was significantly higher in pigeon skeletal muscle compared to that of rat (Table 3). The two non-enzymatic antioxidant defences that were compared showed no rat-pigeon difference in reduced glutathione levels in either the heart or liver, but blood levels of reduced glutathione and plasma uric acid content were significantly higher in the pigeon compared to the rat (Table 3).

**Table 3:** Total antioxidant status, enzymatic (SOD, GPx and CAT) and non-enzymatic antioxidants (GSH, Uric acid) in plasma, heart, pectoral muscle and liver (total tissue and mitochondria) of rats and pigeons.

Parameter	Tissue	Rat	Pigeon	Significance
**Total Antioxidants**				
Total antioxidant status	Plasma	0.8±0.2	1.2±0.1	
Ferric reducing ability	Plasma	0.3±0.0	0.3±0.0	
	Heart	7.0±0.5	7.6±0.3	
	Muscle	0.9±0.1	2.1±0.2	**
	Liver	11.5±1.0	12.6±1.1	
**Non-enzymatic Antioxidants**				
Reduced glutathione	Blood	0.04±0.0	1.1±0.1	*
	Heart	1.5±0.1	1.6±0.1	
	Liver	3.5±0.8	3.3±0.3	
Uric acid	Plasma	11.8±1.6	19.9±1.9	*
**Enzymatic Antioxidants**				
Superoxide dismutase	Plasma	0.6±0.2	0.3±0.1	
	Heart	136.8±23.3	271.5±32.2	*
	Heart mitochondria	94.1±2.7	16.8±6.8	**
	Heart (% in mitochondria)	71.8±9.9	8.4±2.6	**
	Muscle	18.3±1.0	28.2±2.6	**
	Muscle mitochondria	0.1±0.0	0.4±0.1	p = 0.07
	Muscle (% in mitochondria)	0.5±0.2	1.8±0.8	
	Liver	64.4±13.7	75.9±10.7	
	Liver mitochondria	1.6±0.1	0.9±0.3	
	Liver (% in mitochondria)	2.7±0.6	1.3±0.3	p = 0.06
Glutathione peroxidase	Plasma	84.3±12.4	31.6±4.3	**
	Heart	313.6±27.1	169.5±15.8	**
	Heart mitochondria	34.7±12.8	4.6±1.2	**
	Heart (% in mitochondria)	12.0±5.3	2.7±0.6	p = 0.07
	Muscle	659.3±25.2	685.8±37.0	
	Muscle mitochondria	2.3±0.3	1.8±0.3	
	Muscle (% in mitochondria)	0.4±0.0	0.3±0.1	
	Liver	1142.9±159.8	732.2±46.6	*
	Liver mitochondria	19.3±2.8	0.7±0.1	**
	Liver (% in mitochondria)	1.9±0.5	0.1±0.0	**
Catalase	Plasma	124.6±4.4	18.5±2.9	**
	Heart	5469±776	1778±277	**
	Liver	218.6±17.4	34.1±4.9	**

Muscle  =  pectoral muscle. Total antioxidants were measured in plasma using two different assays, the total antioxidant status assay (TAS) and the ferric reducing ability (FRA) assay. Data are shown in mM Trolox equivalents (TAS), mM Fe(II) (FRA in plasma), umol Fe (II)/g tissue (FRA in tissues), u/ml (SOD, GPx, CAT in plasma), u/g tissue (SOD in heart, GPx and CAT in tissues), u/mg tissue (SOD in pectoral muscle and liver), mM (GSH in plasma), nmol/g tissue (GSH in tissues), mg/dl (uric acid). Shown are means ± SEM, n = 6 for rats and n = 8 for pigeons, Significant differences are illustrated through one (p<0.05) or two asterisks (p<0.01). SOD  =  superoxide dismutase, GPx  =  glutathione peroxidase, CAT  =  catalase, GSH  =  reduced glutathione.

Three enzymatic antioxidant defences were compared in most tissues, with catalase activity (CAT) being measured in plasma, heart and liver homogenates, and the other two antioxidant enzymes, superoxide dismutase (SOD) and glutathione peroxidase (GPx), being measured in plasma, the three tissue homogenates, and in the respective mitochondrial preparations. This enabled us to calculate, for both SOD and GPx, the percentage of total tissue antioxidant enzyme activity that is located in the mitochondrial compartment.

Catalase activities in plasma, heart and liver from the rat were significantly greater (3–6 times) than measured for the pigeon tissues. There were no rat-pigeon differences in plasma SOD, total liver SOD, liver mitochondrial SOD, or skeletal muscle mitochondrial SOD. However, the rat had a significantly lower total heart SOD and total skeletal muscle SOD activities than the pigeon. Unlike total heart SOD, the activity of heart mitochondrial SOD was significantly greater (∼6-fold) in the rat compared to the pigeon. This meant that whereas only 8% of the pigeon heart SOD activity was located in the mitochondrial compartment, the corresponding value for the rat was 72%. While there was no rat-pigeon difference in skeletal muscle GPx activity (either with respect to total tissue GPx or mitochondrial GPx), this was not the case for other tissues that were measured. The GPx activities of plasma, heart, heart mitochondria, liver and liver mitochondria were all significantly greater in the rat than the pigeon. The degree of the rat-pigeon differences in mitochondrial GPx activities were much greater (8-28 fold) than the differences observed total GPx activities, which were only 1.6 to 3-fold greater (Table 3).

Total antioxidant status in each tissue correlated with the cumulative activities of the enzymatic and non-enzymatic antioxidants in the respective tissues. For example, plasma enzymatic antioxidant levels were higher, while plasma non-enzymatic antioxidant levels were lower in the rat, resulting in a similar total antioxidant status in the plasma of rats and pigeons. A similar pattern occurred in heart tissue, with rats having a higher total antioxidant status and also more enzymatic antioxidants and in liver, where total antioxidant status as well as SOD and GPx concentrations were similar in both species (Table 3).

A complication in accurately measuring primary ROS production is the potential for mitochondrial enzymes to break down superoxide and hydrogen peroxide before they can be measured. For example, GPx levels in heart mitochondria were very high in the rat compared to the pigeon (Table 3), which might result in H_2_O_2_ breakdown, before H_2_O_2_ leaves the mitochondrial matrix and can be detected by the measurement system. As we were not interested in differences in the absolute levels of superoxide generation, but in differences of the potential for oxidative damage in rats and pigeons, the dismutation of H_2_O_2_ by GPx does not affect the interpretation of the results. However, H_2_O_2_ is also removed non-enzymatically producing hydroxyl radicals during the Fenton reaction [53], but we did not measure these radicals.

### Susceptibility of rat and pigeon membrane lipids to peroxidation

**Figure 3:**
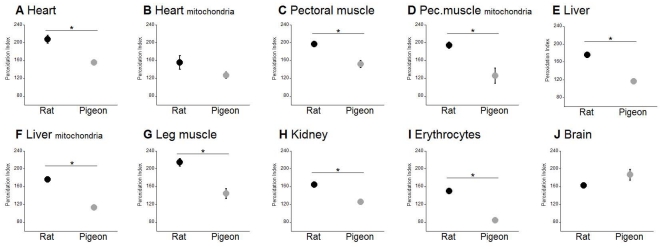
The peroxidation index (PI) of rat and pigeon tissues and mitochondria. The PI describes the susceptibility of membranes to damage by reactive oxygen species. It is calculated taking into account the combination of the relative susceptibilities of different fatty acids to peroxidation. Shown are means ± SEM, n = 6 for rats and n = 8 for pigeons (besides heart mitochondria where n = 3 for rats and n = 5 for pigeons, and brain where n = 3 for rats), Significant differences are highlighted with an asterisk (p<0.01).

The PI for phospholipids (membrane lipids) isolated from seven tissues and three mitochondrial preparations from both species are shown in Fig. 3 (for the actual fatty acid composition data see Table S1). Membrane lipids from all tissues (apart from the brain) and mitochondria of the rat showed a greater susceptibility to peroxidative damage than those from the pigeon. These differences were all statistically significant, except for heart mitochondria, where the lack of significant rat-pigeon difference was likely due to the low sample size (N = 3) for the rat.

### Oxidative damage in rats and pigeons

**Figure 4:**
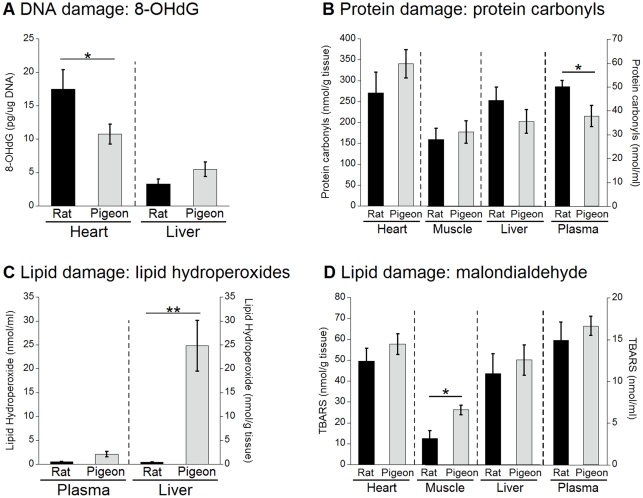
Oxidative damage in rat and pigeon tissues. Products of oxidative damage were measured in rat and pigeon plasma, heart, pectoral muscle and liver. (A) Lipid hydroperoxides, (B) TBARS (thiobarbituric acid reactive substances), (C) 8-OHdG (8-hydroxy-2-deoxy-guanosine) content of mitochondrial DNA, and (D) protein carbonyl content. Shown are means ± SEM, n = 6 for rats and n = 8 for pigeons. Significant differences are highlighted with one (p<0.05) or two asterisks (p<0.01).

The levels of mitochondrial 8-hydroxy-2-deoxy-guanosine (8-OHdG) and protein carbonyls (bio-markers of mitochondrial DNA and protein damage, respectively), as well as TBARS and lipid hydroperoxides (both markers of lipid peroxidation) in tissues from both species are presented in Figure 4. We acknowledge that the TBARS assay is only a crude measure of lipid peroxidation as, besides measuring malondialdehyde, other aldehydes and carbonyl compounds from carbohydrates are detected by this method [54], [55]. Therefore, in the following results and discussion sections the results are presented as TBARS levels and not as malondialdehyde levels.

There were few significant rat-pigeon differences in markers of oxidative damage, but the degree of these differences was relatively small. Rats exhibited significantly higher levels of mitochondrial DNA damage in the heart (Fig 4A), and significantly higher oxidative protein damage to plasma proteins, but not in other tissues (Fig. 4B). Unlike the DNA and protein damage results, lipid hydroperoxide concentrations were significantly higher in liver (but not in plasma) of pigeons compared to rats (Fig. 4C) and TBARS levels were also significantly higher in pigeon skeletal muscle, but showed no difference between rats and pigeons for the other tissues (Fig. 4D).

## Discussion

One of the challenges in interpreting results from studies examining components of the oxidative stress theory in particular species is the limited number of tissues examined and the substantial variation between laboratories. Our experimental design overcomes these limitations, but clearly reveals inherent inconsistencies between expected differences in oxidative stress variables between a long versus short-living species. For example, of the large number of parameters we measured in long-living pigeons and short-living rats, the majority (54%) of these did not differ between the two species. For others, the rat values are significantly higher than the pigeon values (35%), while for the remainder (11%) of the comparisons, the pigeon values are significantly greater than the rat values. It was only the membrane lipid comparisons that showed a consistent pattern of statistically significant differences (all rat PI values > pigeon values). For the other comparisons, mitochondrial ROS production, antioxidants, and oxidative damage, statistically significant differences included cases where rat values exceeded pigeon values, as well as cases where pigeon values were greater than rat values.

Another perspective on the ability of the oxidative stress theory to account for the 7-fold longevity difference between rats and pigeons is to consider the relative size of differences in measured variables between the two species and not just their statistical significance. For this reason, we have plotted “rat value/pigeon value” ratios for the parameters measured. For comparative purposes we have also plotted two lines in this figure; one for a ratio  =  1 (i.e. rat  =  pigeon) and the other when the rat/pigeon ratio  =  7, representing the magnitude of their longevity ratio. For mitochondrial ROS production, antioxidants and oxidative damage categories, we have plotted the direct ratio of each rat/pigeon value, because, in the absence of better knowledge, we have assumed a simple linear relationship between longevity and the particular parameter (e.g. doubling of ROS production might be associated with halving of longevity, etc.). By contrast, for membrane peroxidation index (PI) category, the values plotted are (rat/pigeon)^3^. This transformation is justified because, unlike the other categories, we already know that the relationship between membrane PI and longevity is not simple and linear. In a wide variety of birds and mammals, the PI is proportional to the −0.30 power of MLSP for skeletal muscle phospholipids, and the −0.36 power of MLSP for liver mitochondrial phospholipids [56]. If we assume for tissues in general that PI is proportional to MLSP^−0.33^, then such a relationship can be transformed to MLSP being inversely proportional to PI^3^.

**Figure 5:**
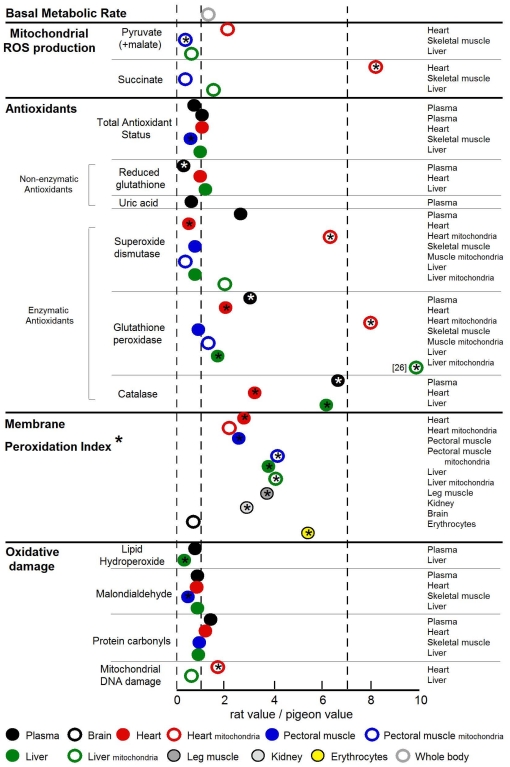
Rat/Pigeon ratios for all parameters determined in this study. For comparative purposes, two lines are plotted in this figure, one line at a ratio  =  1 (i.e., rat  =  pigeon) and the other when the ratio rat/pigeon  =  7 (representing the 7-fold longevity difference of rats and pigeons). For all parameters besides membrane PI we have plotted the direct ratio of each rat/pigeon value, whereas for the PI comparison the values plotted are (rat/pigeon)^3^ (see discussion section for details). Significant differences between rats and pigeons are highlighted with an asterisk (p<0.05).

The picture that emerges from Figure 5 is, we believe, illuminating. Of all the variables measured, only some of the enzymatic antioxidants and most membrane PI values were of consistently higher magnitude in rats compared to pigeons. Total antioxidant status and non-enzymatic antioxidants are essentially the same in rats and pigeons, but the enzymatic antioxidants, especially mitochondrial, suggest that rats experience a much greater degree of oxidative stress *in vivo* than do pigeons. This is especially the case for the peroxidases (GPx and CAT). By contrast, our *in vitro* measurements of mitochondrial ROS production do not support this interpretation, with only heart mitochondria showing greater rates of ROS production in rats compared to pigeons, but only with succinate as a substrate. It is noteworthy that the commonly expressed conclusion that rat mitochondria have a greater superoxide production than pigeon mitochondria is to a large extent based on results obtained from heart mitochondria [15], [16], [21], [22], [33], with only two studies showing a higher ROS production in rat brain, liver, lung and kidney mitochondria [20], [57].Our measurements raise doubts that rat tissues generally have higher rates of mitochondrial ROS production than those of pigeons, but the higher enzymatic antioxidant content of rats compared to pigeons suggests that rat tissues *in vivo* likely experience greater oxidative stress than pigeon tissues.

The apparent contradiction between the absence of differences in mitochondrial ROS production in most rat tissues compared to pigeons, despite having higher antioxidant activities, raises concern as to how well *in vitro* measurements approximate *in vivo* conditions. It is possible that the saturating levels of substrates that we and most other studies used in *in vitro* determinations of ROS production produce different outcomes than when physiological substrate concentrations are provided, as found in a multi-species study by Brown et al. [9].

There is considerable variation in literature values of mitochondrial ROS production. Early assertions that 1–4% of mitochondrial electron transport *in vivo* is diverted (“leaked”) to superoxide production are based on unrealistic assumptions that have been disproven [50]. More realistic assumptions suggest that the value is more likely to be about 0.15% [33], which corresponds with the values we have obtained for rat and pigeon mitochondria from all three tissues (Figure 2). We have no evidence that the rate of electron leakage is greater in rats than pigeons, except when succinate is the only substrate provided. Rotenone (the complex I inhibitor) diminishes superoxide generation showing that a large proportion of superoxide is generated at complex I during “reverse electron transport” (Fig.1). The physiological relevance of this condition, however, has been questioned [58]. Extrapolation from *in vitro* measurements to the *in vivo* situation is also difficult because little is known about changes in mitochondrial superoxide production in response to physiologically relevant changes in substrate supply and energy demand in intact cells [50].

The lack of interspecific differences in levels of oxidative-damage products between rats and pigeons is contrary to predictions of the oxidative stress theory of aging. Inferring oxidative stress rates from endogenous levels of particular oxidative damage products is, however, problematic. Such products are rapidly repaired or removed, making oxidative stress biomarkers representative of steady-state levels only. We found the levels of a variety of biomarkers to be very similar in rats and pigeons, indicating that these markers are not necessarily a good measure of oxidative stress *in vivo*. This adds to a growing body of evidence that protein carbonyls and other glycated protein adducts are not appropriate markers of aging-related damage [26], [28], [59], [60]. Giron-Calle et al. (1997) have previously demonstrated the rapidity of membrane lipid turnover in the presence of oxidative stress. The induction of lipid peroxidation in rat hepatocytes resulted in increased TBARS levels, but had no effect on membrane fatty acid composition [61]. The rapid deacylation-reacylation processes highlight the importance of repair and removal mechanisms during intense oxidative stress. Mechanisms that remove lipid peroxidation products include the activity of peroxidases such as GPx (more specifically GPx 4), which has specificity to a variety of hydroperoxides, including lipid hydroperoxides [62], [63]. The low concentration of lipid hydroperoxides we measured in rat liver is likely due to the rapid rate of break-down by the high mitochondrial GPx levels in the rat.

Another challenge in using oxidative-damage products to identify sources of oxidative stress is their interactive nature. Whereas DNA damage is a consequence of the attack of primary ROS (mainly hydroxyl radicals), carbonyls can be introduced into proteins either through direct oxidation (through primary ROS), via reactions with lipid peroxidation products (4-hydroxy-2-nonenal, malondialdehyde) or in glycation and glycoxidation reactions [64], [65], [66]. Lipid hydroperoxides are created from all polyunsaturated fatty acids, whereas malondialdehyde is the main product from the peroxidation of arachidonic acid [67]. As primary ROS lead to a chain reaction producing a large variety of secondary ROS, which, in turn, damage particular molecules, evaluating particular markers does not reveal the actual levels or sources of oxidative stress. Even when differences in oxidative-damage products are found, these do not consistently explain large lifespan differences between species. Naked mole-rats, with a maximum lifespan of more than 28 years, show higher levels of lipid peroxidation, DNA and protein oxidative damage in comparison to mice (MLSP 5 years), and these differences were evident from a very young age [68], [69]. Similarly high levels of oxidative damage are also apparent in other long-living species, such as birds and bats [28], [70].

Of all our measurements, the most consistent difference we observed between rats and pigeons was the peroxidation index of membrane lipids. Our results confirm earlier reports of rat-pigeon differences in the fatty acid composition of mitochondrial membranes from heart [25], [71] and liver [26], [71], and extend this finding to skeletal muscle mitochondria. We further found that this difference in mitochondrial membrane composition applies to total phospholipids of the heart, liver and skeletal muscle as well as other tissues. Although the rats and pigeons were fed different diets, this is unlikely to influence membrane composition. At least in rats, membrane fatty acid composition is a regulated variable and relatively little affected by diet fatty acid composition [72], [73] and specifically membrane PI is constant irrespective of wide variation in diet PI (Abbott and Hulbert, unpublished observations). Other evidence that membrane composition appears to be a regulated variable comes from the observation that mice strains that differ in longevity have different membrane composition despite being fed the same diet and maintained under identical animal housing for their entire lives [74]. We assume that a similar situation exists between diet and membrane composition for pigeons.

Membrane lipids are the source of a large group of powerful lipid-based ROS (which we will collectively call secondary ROS) formed from the action of primary ROS (superoxide and hydrogen peroxide) on membrane polyunsaturated fatty acids. These powerful lipid-based ROS are continually formed during lipid peroxidation and are responsible for the autocatalytic positive-feedback nature of this process. They also are responsible for much of the oxidative damage to other non-lipid bio-molecules, including proteins [66]. The importance of membrane lipid composition lies in the fact that if we compare two membranes that differ in their fatty acid composition and PI (and consequently their susceptibility to peroxidative damage) under conditions of identical challenge from primary ROS, it is the more susceptible membrane (the one with the higher PI) that will produce a greater degree of oxidative stress. In other words, it is the level of total ROS production (primary ROS + secondary ROS) that is responsible for determining the level of oxidative stress and not solely superoxide production. Thus the lack of a difference in mitochondrial superoxide production does not necessarily mean a lack of a difference in the level of oxidative stress and conversely, differences in the level of oxidative stress do not necessarily require differences in mitochondrial superoxide production.

There is evidence that rats have higher secondary ROS production rates than pigeons, with consequently higher levels of oxidative-damage products. Pamplona et al. [25] have found that the low PI of heart mitochondrial lipids in pigeons results in both a low level of lipid peroxidation and reduced lipoxidative damage to proteins relative to rats. Importantly, the low peroxidative susceptibility of pigeon mitochondrial phospholipids was shown both *in vivo* and *in vitro* [25], [26], [27], demonstrating the resistance of pigeon membranes to oxidative insults. We suggest that high PI of rat membranes act as a ROS amplifier by leading to the production of harmful levels of secondary lipid-based ROS, and therefore to more oxidative damage. This might be verified using recently developed methods to determine lipid peroxidation rates. Stanley Hazen and his colleagues describe an indirect way of measuring the production of secondary, lipid-based ROS. The peroxidation of polyunsaturated fatty acids within membranes leads to the enzymatic cleavage of the damaged products, leaving a truncated fatty acid terminating in an aldehyde or carboxylic acid group. This modified fatty acid is able to move from the interior of the membrane into the aqueous environment, forming “lipid whiskers” on the membrane surface [75]. The quantification of those lipid whiskers within biological membranes might be a helpful tool to determine rates of secondary ROS production.

Given the lack of correspondence between the rat-pigeon ratios of most variables we measured and their 7-fold difference in MLSP (Fig. 5), we conclude that the substantive differences in mitochondrial ROS production reported previously for these species was due to the selection of heart muscle to source the mitochondria and the decision to provide succinate as a substrate. Had mitochondria been harvested from other tissues or inferences been drawn from comparison of levels of randomly chosen antioxidants, then very different conclusions regarding differences in oxidative stress between these species would have been reached. Another consideration raised by our results is whether the oxidative stress theory of aging requires that all tissues of animals with differing MLSP show proportionately different levels of oxidative stress. In other words, are differences in longevity driven mainly by system-wide failures, or simply by failure of a key organ or system? Additionally, the consistently higher PI of rat membranes compared to pigeons raises the possibility that differences in MLSP between these species may be related to significantly higher total ROS production in rats due to their higher rates of secondary ROS formation by lipoxidation processes.

The potential importance of membrane fatty acid composition in the determination of longevity is supported by other recent reports on membrane composition in other exceptionally long-living mammals (e.g. naked mole-rats and echidnas) [76], [77]. It is also supported by two other recent genetic studies. One of these examining *C.elegans* mutants encompassing a 10-fold longevity difference showed that modulation of lipid biosynthesis and membrane composition contributed to stress resistance and longevity of these worms [78]. They showed a similar relationship between membrane PUFA content (and peroxidation index) and maximum lifespan in *C.elegans* to that previously reported for birds and mammals [79]. The other genetic study examined the evolutionary selection of candidate genes for longevity in mammals and identified those involved with lipid composition to be one of two genes strongly conserved among mammals with high MLSP [80]. Further comparative studies involving bird species with very different MLSPs will confirm whether relative decreases in membrane PI are a general feature among long-lived endothermic vertebrates.

## Supporting Information

Table S1 Fatty acid composition of whole tissue (liver, pectoral muscle, leg muscle, heart, kidney, brain and erythrocytes) and mitochondrial (liver and pectoral muscle) phospholipids (mol %) of pigeons and rats. Shown are means ± SEM; included in each column is the number of animals used from each species and tissue. SFA  =  saturated fatty acids, MUFA  =  monounsaturated fatty acids, PUFA  =  polyunsaturated fatty acids, UI  =  unsaturation index, PI  =  peroxidation index.
(DOC)
