# Correction: The Long Life of Birds: The Rat-Pigeon Comparison Revisited

**DOI:** 10.1371/annotation/cba5e1ce-429f-4b46-8499-d56a55a944dc

**Published:** 2011-11-10

**Authors:** Magdalene K. Montgomery, A. J. Hulbert, William A. Buttemer

The correct first affiliation is: School of Biological Sciences, University of Wollongong, Wollongong, New South Wales, Australia. 

