# Supplementary material for: The Long Life of Birds: The Rat-Pigeon Comparison Revisited
Source: PLoS One. 2011 Aug 31;6(8):e24138. doi: 10.1371/journal.pone.0024138 (PMC3164121; doi:10.1371/journal.pone.0024138)
Supplement: Table S1 — Fatty acid composition of whole tissue (liver, pectoral muscle, leg muscle, heart, kidney, brain and erythrocytes) and mitochondrial (liver and pectoral muscle) phospholipids (mol %) of pigeons and rats. Shown are means ± SEM; included in each column is the number of animals used from each species and tissue. SFA = saturated fatty acids, MUFA = monounsaturated fatty acids, PUFA = polyunsaturated fatty acids, UI = unsaturation index, PI = peroxidation index. (DOC) [file pone.0024138.s001.doc]

Fatty acid composition of whole tissue (liver, pectoral muscle, leg muscle, heart, kidney, brain and erythrocytes) and mitochondrial (liver and pectoral muscle) phospholipids (mol %) of pigeons and rats. Shown are means ± SEM; included in each column is the number of animals used from each species and tissue. SFA = saturated fatty acids, MUFA = monounsaturated fatty acids, PUFA = polyunsaturated fatty acids, UI = unsaturation index, PI = peroxidation index

**Heart Pectoral muscle**

Tissue Mitochondria Tissue Mitochondria

Rat Pigeon Rat Pigeon Rat Pigeon Rat Pigeon

number animals 6 7 4 5 6 8 6 8

C16:0 13.7±0.4 12.3±1.5 16.1±2.4 15.3±1.9 23.1±0.3 6.8±1.8 16.6±1.1 7.9±1.0

C16:1 n7 0.2±0.0 0.5±0.0 0.8±0.3 0.9±0.1 0.5±0.0 0.4±0.0 0.5±0.1 0.3±0.0

C18:0 21.5±0.9 19.3±0.6 21.9±0.5 23.6±1.2 15.2±0.2 24.7±0.6 19.2±1.0 22.8±3.1

C18:1 n9 4.0±0.3 5.3±0.4 6.6±0.5 6.2±0.4 5.3±0.1 5.0±0.5 4.9±0.5 4.5±0.5

C18:1 n7 4.5±0.1 1.9±0.1 3.6±0.7 2.2±0.2 3.1±0.1 2.9±0.2 3.2±0.2 2.3±0.3

C18:2 n6 19.1±1.4 30.8±1.0 17.7±2.3 22.2±2.1 19.4±0.7 33.4±1.6 21.8±1.3 26.0±3.6

C18:3 n3 0.1±0.0 0.5±0.1 0.2±0.1 0.3±0.0 0.2±0.0 0.3±0.0 0.3±0.1 0.2±0.0

C20:0 0.2±0.0 0.6±0.0 0.3±0.1 0.3±0.0 0.1±0.0 0.3±0.0 0.1±0.0 0.2±0.0

C20:1 n9 0.1±0.0 0.1±0.0 0.3±0.1 0.2±0.1 0.1±0.0 0.1±0.0 0.2±0.1 0.1±0.0

C20:2 n6 0.2±0.0 0.1±0.0 0.2±0.0 0.1±0.0 0.2±0.0 0.1±0.0 0.1±0.0 0.1±0.0

C20:4 n6 20.5±0.8 21.2±0.8 14.2±0.6 18.9±0.8 15.0±0.3 19.2±1.8 13.5±0.7 17.8±2.5

C20:5 n3 0.1±0.0 0.5±0.1 0.2±0.2 0.5±0.0 0.2±0.0 0.4±0.1 0.3±0.0 0.5±0.1

C22:0 0.1±0.0 0.1±0.0 0.2±0.0 0.1±0.0 0.1±0.0 0.1±0.0 0.1±0.0 0.1±0.0

C22:5 n3 2.3±0.1 2.3±0.1 1.5±0.3 1.3±0.2 2.8±0.1 2.9±0.2 2.2±0.2 1.8±0.3

C22:6 n3 10.2±1.0 1.9±0.2 7.8±1.2 1.7±0.2 11.1±0.8 1.7±0.2 11.7±1.0 1.3±0.2

Σ SFA 35.8±1.0 32.9±1.9 41.9±3.9 41.1±1.8 38.9±0.3 31.4±2.7 38.2±2.2 31.4±4.2

Σ unsaturated 64.2±1.0 67.1±1.9 58.1±3.9 58.9±1.8 61.1±0.3 68.6±2.7 61.8±2.2 56.6±7.8

Σ MUFA 9.5±0.4 8.2±0.4 14.4±0.6 12.8±1.4 9.7±0.1 8.8±0.6 9.4±0.6 7.8±1.1

Σ PUFA 54.8±1.3 58.8±1.8 43.7±4.0 46.1±2.9 51.4±0.3 59.8±2.7 52.4±2.0 48.9±6.7

Σ n-6 PUFA 42.0±0.8 53.4±1.6 34.0±2.4 42.2±2.6 36.8±1.0 54.2±2.5 37.7±2.0 44.9±6.2

Σ n-3 PUFA 12.8±1.1 5.3±0.4 9.6±1.7 3.7±0.4 14.5±0.8 5.5±0.4 14.6±1.2 3.9±0.5

UI 212.9±6.5 187.8±5.8 170.4±14.4 156.8±7.1 200.8±2.2 187.4±8.8 200.1±6.9 155.1±22.1

PI 207.4±8.9 155.0±5.4 155.3±15.4 126.4±6.8 196.5±4.3 151.6±8.4 194.1±7.3 125.6±17.

**Liver Leg muscle Kidney**

Tissue Mitochondria Tissue Tissue

Rat Pigeon Rat Pigeon Rat Pigeon Rat Pigeon

number animals 6 8 6 8 6 8 6 8

C16:0 21.0±1.0 11.0±0.6 18.8±0.8 9.5±1.6 23.3±0.8 9.8±1.2 21.0±0.5 12.7±1.8

C16:1 n7 1.0±0.2 0.5±0.1 1.1±0.2 0.5±0.1 0.5±0.0 0.8±0.3 0.5±0.1 0.4±0.0

C18:0 19.0±0.7 28.6±1.4 18.1±0.4 28.1±1.1 15.4±0.2 24.3±1.3 19.0±0.3 24.4±1.3

C18:1 n9 4.3±0.2 8.9±1.0 4.7±0.3 10.3±0.8 4.3±0.2 8.2±1.8 6.5±0.2 9.1±1.3

C18:1 n7 3.7±0.2 2.0±0.1 3.5±0.1 1.9±0.1 3.3±0.2 2.5±0.2 2.2±0.1 2.6±0.6

C18:2 n6 13.5±0.8 27.9±0.6 16.7±0.6 29.3±1.2 16.4±1.5 28.7±1.8 10.3±0.7 25.0±4.1

C18:3 n3 0.1±0.0 0.2±0.0 0.2±0.0 0.3±0.1 0.2±0.0 0.3±0.0 0.1±0.0 0.2±0.1

C20:0 0.1±0.0 0.2±0.0 0.1±0.0 0.1±0.0 --- 0.5±0.1 0.2±0.0 0.5±0.0

C20:1 n9 0.2±0.0 0.2±0.0 0.2±0.0 0.1±0.0 0.1±0.0 0.1±0.0 0.1±0.0 0.3±0.1

C20:2 n6 0.4±0.0 0.3±0.0 0.1±0.0 0.2±0.0 0.2±0.0 0.3±0.2 0.3±0.0 0.3±0.1

C20:4 n6 26.1±0.8 13.6±0.3 25.7±0.7 13.3±0.3 17.1±0.6 17.1±1.5 31.7±0.5 15.4±1.3

C20:5 n3 0.4±0.0 0.4±0.1 0.4±0.0 0.4±0.1 0.3±0.0 0.6±0.1 0.3±0.0 0.8±0.1

C22:0 0.1±0.0 0.1±0.0 --- 0.1±0.0 0.1±0.0 0.2±0.0 0.3±0.0 0.4±0.1

C22:5 n3 1.3±0.1 0.8±0.1 1.2±0.1 0.7±0.1 3.1±0.1 2.7±0.3 0.4±0.0 0.6±0.1

C22:6 n3 5.1±0.3 2.3±0.5 5.1±0.3 2.1±0.4 12.4±1.4 2.1±0.3 2.2±0.1 2.7±1.2

Σ SFA 41.5±0.6 40.3±1.5 38.6±0.6 38.5±0.8 39.2±0.9 33.6±3.6 42.9±0.4 39.0±1.3

Σ unsaturated 58.5±0.6 59.7±1.6 61.4±0.6 61.5±0.7 60.8±0.9 66.4±3.6 57.1±0.4 61.0±1.3

Σ MUFA 9.7±0.5 11.9±1.2 10.0±0.5 13.2±0.8 8.7±0.2 12.3±2.1 10.2±0.2 13.8±3.1

Σ PUFA 48.8±0.7 47.8±0.6 51.4±0.6 48.3±1.1 52.1±0.9 54.1±3.6 47.0±0.5 47.2±3.6

Σ n-6 PUFA 41.7±1.1 43.8±0.6 44.3±0.9 44.5±1.4 36.0±2.0 48.1±3.8 43.9±0.5 42.7±4.6

Σ n-3 PUFA 7.0±0.4 3.8±0.4 6.8±0.4 3.5±0.3 16.0±1.5 5.8±0.5 3.0±0.1 4.4±1.0

UI 187.9±1.7 151.0±4.0 192.3±1.1 151.5±1.7 211.8±4.7 177.9±11.5 180.8±1.1 158.3±3.7

PI 175.4±2.1 116.5±4.1 175.9±1.2 113.2±2.2 214.5±8.6 144.0±11.2 164.2±1.5 125.6±4.9

**Brain Erythrocytes**

Tissue Mitochondria

Rat Pigeon Rat Pigeon

number animals 4 8 6 8

C16:0 23.2±0.6 24.7±2.2 28.7±0.8 15.1±0.7

C16:1 n7 0.4±0.0 0.6±0.1 0.5±0.1 0.6±0.

C18:0 20.2±0.2 21.0±1.1 13.5±0.4 23.1±0.7

C18:1 n9 18.7±0.3 11.0±1.1 7.0±0.4 10.9±0.7

C18:1 n7 3.7±0.2 4.8±0.6 3.1±0.1 1.7±0.1

C18:2 n6 0.6±0.0 3.6±1.6 10.9±0.5 33.4±0.8

C18:3 n3 --- --- 0.2±0.0 0.1±0.0

C20:0 0.4±0.0 0.2±0.1 0.1±0.0 0.2±0.0

C20:1 n9 1.5±0.2 0.3±0.2 0.2±0.0 0.4±0.0

C20:2 n6 0.1±0.0 0.1±0.0 0.4±0.0 0.6±0.0

C20:4 n6 9.8±0.5 11.4±1.0 23.4±0.4 5.7±0.2

C20:5 n3 0.1±0.0 0.1±0.0 0.3±0.0 0.2±0.0

C22:0 0.4±0.0 0.2±0.1 0.3±0.0 0.3±0.0

C22:5 n3 0.2±0.0 0.3±0.1 1.9±0.1 1.0±0.0

C22:6 n3 13.1±0.3 13.4±1.9 2.5±0.2 1.6±0.3

Σ SFA 45.4±0.6 46.7±2.0 45.5±0.7 39.4±0.3

Σ unsaturated 54.6±0.6 53.3±2.0 54.5±0.7 60.6±0.3

Σ MUFA 26.9±1.1 18.6±2.6 12.0±0.4 16.0±0.7

Σ PUFA 27.7±0.5 34.8±2.1 42.6±0.8 44.6±0.7

Σ n-6 PUFA 14.3±0.6 20.7±3.8 37.4±0.7 41.5±0.8

Σ n-3 PUFA 13.4±0.3 13.8±1.8 5.0±0.4 3.0±0.3

UI 162.8±0.9 179.2±5.2 166.2±3.1 130.0±1.3

PI 162.3±2.2 186.3±12.5 149.4±3.6 84.0±2.0
